# Atypical clinical features of post COVID‐19 mucormycosis: A case series

**DOI:** 10.1002/cre2.743

**Published:** 2023-05-01

**Authors:** Hassanien A. Al‐jumaily, Auday M. Al‐Anee, Ahmed F. Al‐Quisi

**Affiliations:** ^1^ Oral and Maxillofacial Surgery Department, College of Dentistry University of Baghdad Baghdad Iraq; ^2^ Al‐Shaheed Gazi Al‐Hariri Teaching Hospital Medical City Baghdad Iraq; ^3^ Al‐Kindy Teaching Hospital Baghdad Iraq

**Keywords:** COVID‐19, diabetes mellitus, mucormycosis

## Abstract

**Objectives:**

This case series aims to evaluate patients affected with post COVID‐19 mucormycosis from clinical presentation to surgical and pharmacological treatment to improve the disease prognosis.

**Material and Methods:**

This case series was conducted at a specialized surgery hospital in Baghdad Medical City for over 10 months. Fifteen cases who had mild to severe COVID‐19 infections followed by symptoms similar to aggressive periodontitis, such as mobility and bone resorption around the multiple maxillary teeth, were included in this case series.

**Results:**

All patients did not receive COVID‐19 vaccination; seven had a history of diabetes mellitus type 2, another five patients had a history of diabetes‐like syndrome during the COVID‐19 infection, and the remaining three patients had no history of any systemic diseases. No intracranial involvement was seen in all patients, and bilateral sinus involvement was seen in three patients.

**Conclusion:**

Being highly suspicious of all patients affected with COVID‐19 is highly recommended to avoid the complications of the late diagnosis of mucormycosis. In addition, our knowledge and methods in diagnosing and treating classical mucormycosis should be modified regarding post COVID‐19 mucormycosis.

## 1 INTRODUCTION

The coronavirus disease of 2019 (COVID‐19) is a pandemic affecting the whole world, which presents significant variation in symptoms. One‐third of patients with COVID‐19 are asymptomatic while in the symptomatic two‐thirds, 81% have mild to moderate symptoms, 14% develop severe symptoms, and 5% suffer from life‐threatening symptoms (Oran & Topol, 2021).

Despite the innovation of vaccines for COVID‐19, which heralds the return of life to normal, the disease still surprises us with new challenges, including mucormycosis (Almas et al., 2021).

Mucormycosis represents a rare acute infection caused by a mucorales, a saprophytic aerobic fungus with great affinity to the paranasal sinuses. It rarely affects otherwise healthy people (Deeplata et al., 2021; Said Ahmed et al., 2021). The most common presentation of mucormycosis is orbital and maxillary cellulitis with mobility of the teeth in the affected maxillary segment (Nambiar et al., 2021; Sharma et al., 2021). Traditionally, mucormycosis starts in the nose and paranasal sinuses of patients with immunocompromising diseases, especially uncontrolled diabetes mellitus, which rarely affect healthy people (Morduchowicz et al., 1986).

Patients with phagocytic dysfunction caused by neutropenia, ketoacidosis, or high serum iron levels are at risk of developing mucormycosis. The innate immune system is enough to eradicate the infection in healthy patients except for those with severely contaminated exposed wounds (Bitar et al., 2009).

The prevalence of this disease had increased in the last few decades due to the increased life span of immunocompromised patients and improved diagnostic methods. However, the incidence of reported cases in the pre COVID‐19 era was still low in developed countries, with blood‐related malignancies as the main predisposing factor. In contrast, uncontrolled diabetes is responsible for increasing mucormycosis in developing countries (Bitar et al., 2009; Skiada et al., 2018).

Interestingly, Marchand et al. (2020) reported the first case of what is called diabetes‐like symptoms in a patient who presented with diabetes mellitus 1 month after being infected with COVID‐19, despite being normoglycemic in the initial hospitalization (Marchand et al., 2020).

Developing the post COVID‐19 diabetes‐like symptoms was thought to be related to the autoimmune mechanism initiated by the cytokine storm in genetically susceptible individuals or due to temporary loss of the β‐cell function with the increased angiotensin‐II enzyme. These patients were classified as having Type 1 diabetes (Roberts et al., 2021).

However, the available data regarding the management and prognosis of this disease were still obtained from case reports and case series because of the impossibility of performing a large randomized controlled trial with such disease (Deeplata et al., 2021; Morduchowicz et al., 1986). Because the number of Rhinocerebral Mucormycosis cases worldwide before the COVID‐19 pandemic was infrequent, diagnosis and treatment of such cases may become difficult for the general practitioner (Song et al., 2020).

This case series reviewed 15 patients who had mild to severe COVID‐19 infections, followed by symptoms similar to aggressive periodontitis, such as mobility and bone resorption around multiple maxillary teeth. In these patients, one side of the upper jaw usually moved as one segment, and histopathological examinations proved the diagnosis of mucormycosis.

## 2 PATIENTS AND METHODS

The case series was conducted at a specialized surgery hospital in Baghdad Medical City for over 10 months. All patients with a history of COVID‐19 infection presented or were referred to the maxillofacial consultation clinic due to recurrent episodes of facial swelling and sinusitis with mobility of teeth on the affected side of the maxilla.

The case series was ethically approved by the Research Ethics Committee of the College of Dentistry, University of Baghdad (Project No. 633122).

Intraoral examination revealed painless aggressive periodontitis with hypermobile teeth and swollen bright red mucosa. A little or no gingival recession involving all teeth on the affected side (right or left) was also noted.

A cone beam computed tomography (CBCT) revealed bony resorption around the affected side of the maxillary teeth, with obliteration of the maxillary and ethmoidal paranasal sinuses on the same side. Many patients had previous periodontic or endodontic treatments for these teeth but with no noted benefit.

Laboratory investigations regarding the level of HbA1c showed that

**Figure 1:**
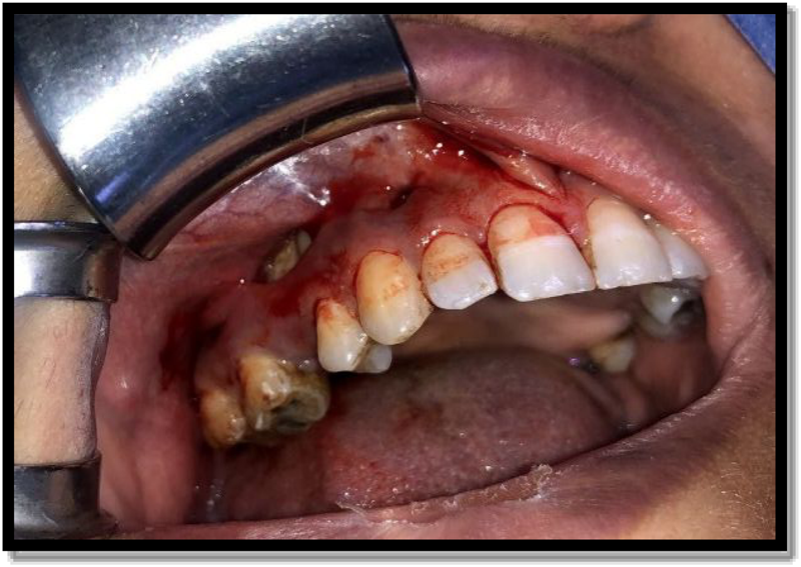
Normally attached mucosa overlying hypermobile maxillary teeth with abnormal yellowish bone from the incision made in the buccal vestibule for decompression of buccal space cellulitis.

Drainage for decompression of the buccal space cellulitis was done in some cases and revealed yellowish bone with total resorption of the buccal bone plate over the affected maxillary teeth; no bleeding was elicited after scratching the affected bone, hence, raising the suspicion about the diagnosis (Figure 1).

A biopsy was taken from the affected bone and sent for histopathological examination. Patients were then prepared for curettage surgery once the histopathological report confirmed the diagnosis of mucormycosis.

**Figure 2:**
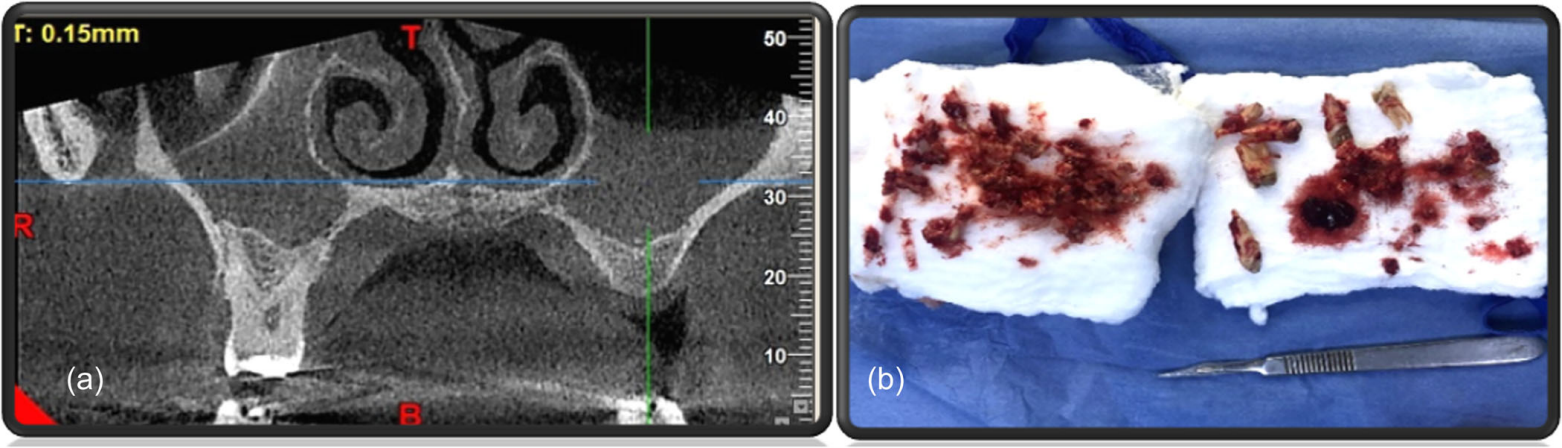
(a) CBCT coronal section showed an obliterated right maxillary air sinus and fluid collection on the left side. (b) Extraction and curettage of the affected teeth and bone on the right side. Suturing with 3/0 black silk sutures to approximate and hold the iodoform pack in place.

The affected teeth were extracted with curettage of the bone, maxillary sinus, copious irrigation with normal saline, and then wound packed with iodoform gauze (Figure 2).

The iodoform pack was changed daily with irrigation of the wound done three times per day, using normal saline mixed with hydrogen peroxide 10:1 for 2 weeks.

This surgical intervention was followed by administration of meropenem powder injected 1 g twice daily, liposomal amphotericin B 5 mg/kg (slow infusion), and metronidazole 500 mg three times daily for the first eight patients.

The subsequent two patients were treated still with the surgical intervention but with meropenem powder 1 g twice daily and metronidazole 500 mg three times daily only.

**Figure 3:**
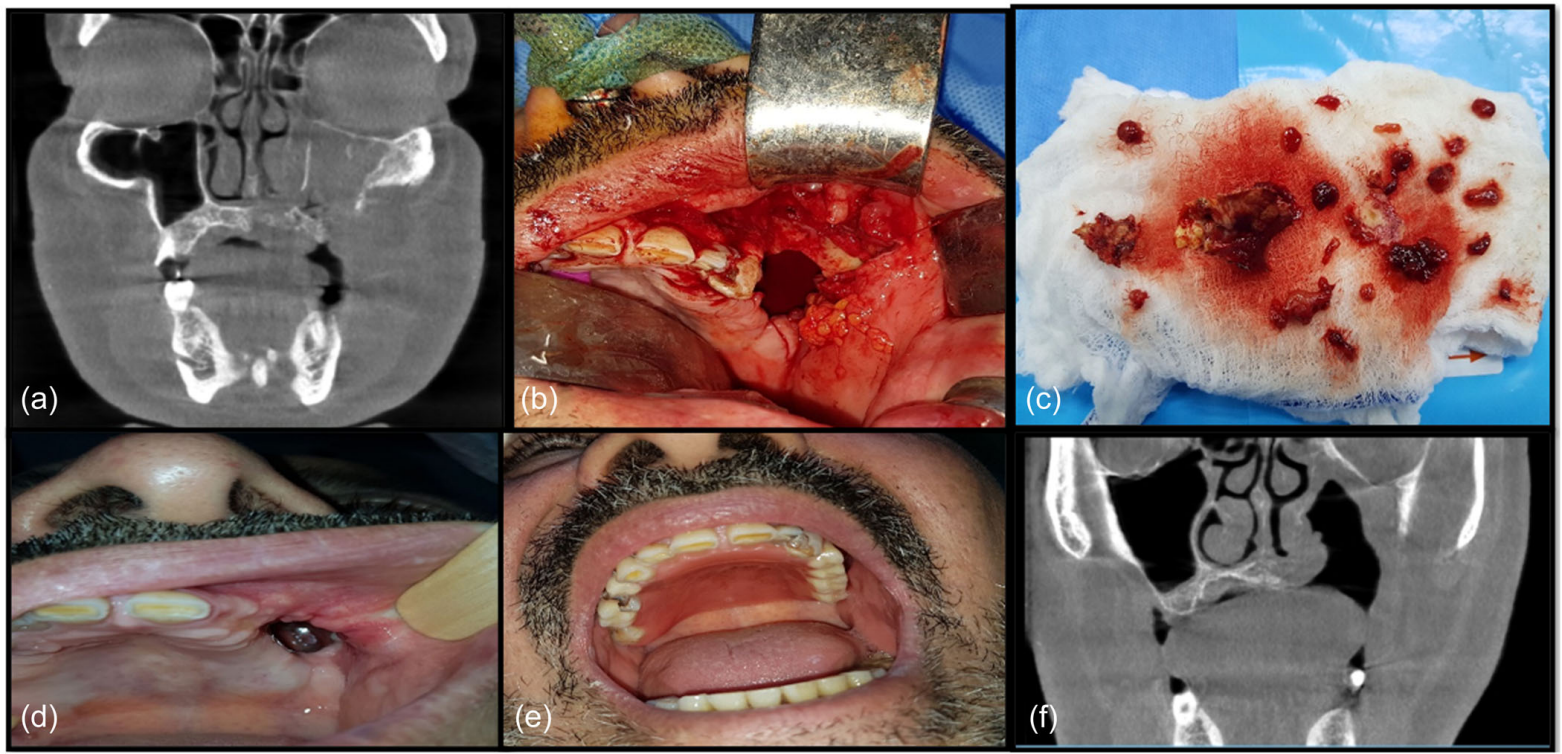
(a) CBCT coronal view showed bilateral obliterated left maxillary sinus air cavity. (b) Surgical removal of the affected teeth and curettage of the affected sinus cavity. (c) Specimens sent for histopathological examination. (d) Clinical appearance of the maxillary defect after 10 months. (e) Patient wearing the obturator. (f) CBCT coronal view after 10 months of follow‐up showed clear sinus cavity with good response to mechanical debridement and antibiotic therapy.

The reason for not giving amphotericin in the remaining two cases is their imprecise primary histopathological reports, although slide revision by an oral pathologist confirmed the diagnosis of mucormycosis. At that time, both patients had complete uneventful healing. Ten months of follow‐up of these cases showed no recurrence (Figure 3).

**Figure 4:**
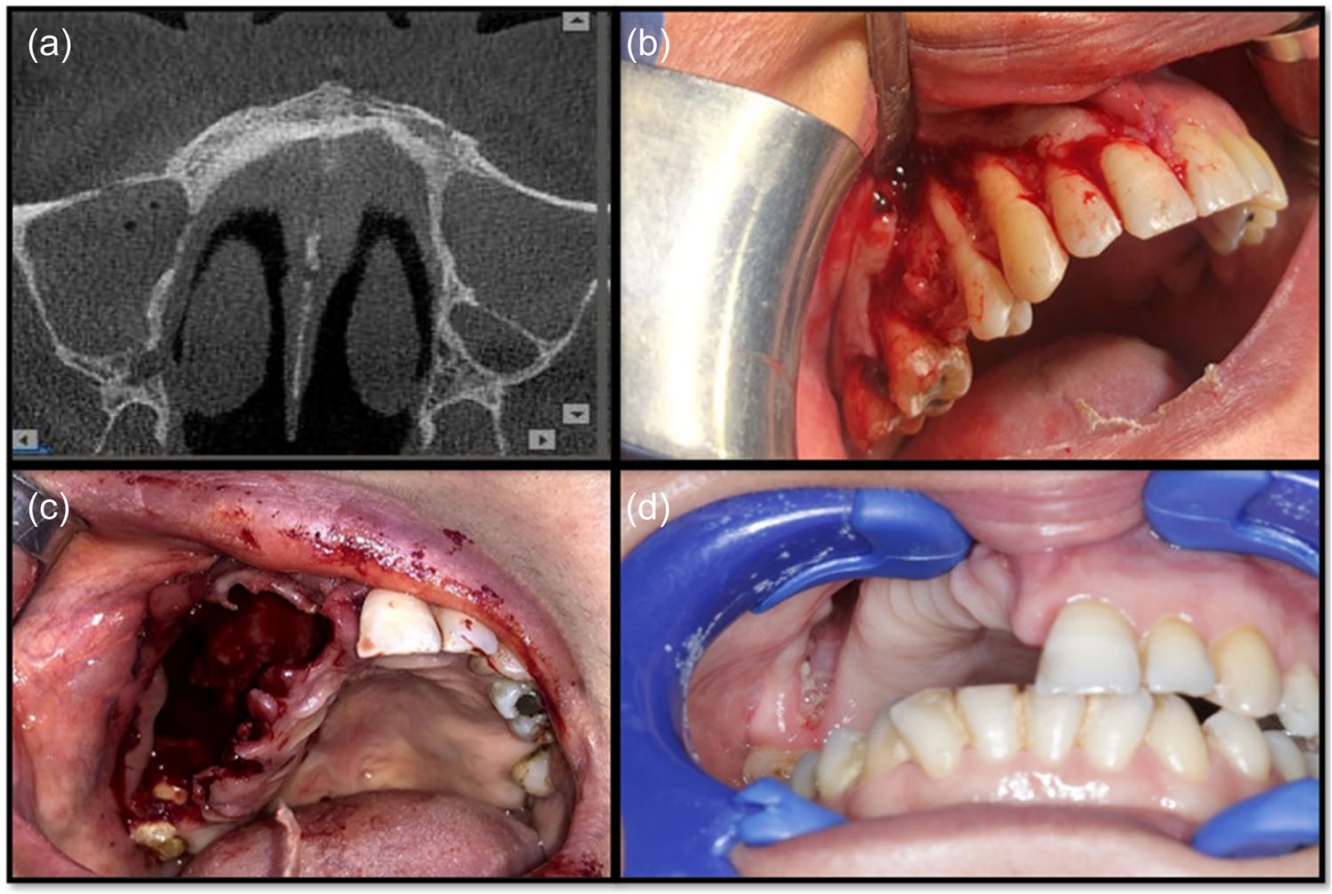
(a) An axial view of CBCT showed obliterated maxillary air sinuses on both sides. (b) Severe bone resorption affecting the buccal bone plates on the right side of the maxilla. (c) Surgical extraction and curettage of the affected teeth and bone. (d) Complete re‐epithelization with no disease recurrence after 4 months of follow‐up.

In the last five cases, liposomal amphotericin B was changed from 5 to 1.5 mg/kg. Patients had good healing with no complications seen during the follow‐up period (Figure 4).

## 3 RESULTS

Fifteen patients were included in this case series; all of them were examined in the oral and maxillofacial surgery consultation clinic in Baghdad Medical City from July 2021 to June 2022.

All patients in this case series were living in Baghdad City withage ranging from 35 to 78‐year‐old, mean age of (57.33) ± 11.6. The average time from developing symptoms of facial swelling and seeking consultation at the clinic was 2 months.

Patients reported that facial swelling started between the 1st and 2nd week after resolution of from COVID‐19 symptoms.

All patients did not receive COVID‐19 vaccination; seven of them had a history of diabetes mellitus type 2, five had a history of diabetes‐like syndrome during the COVID‐19 infection, and the remaining three patients had no history of any systemic diseases.

From history, eight patients developed a severe COVID‐19 infection, and was treated according to the protocol consisting of remdesivir, meropenem, or ceftriaxone in addition to the supportive therapy of vitamins C, D, and zinc and paracetamol as an antipyretic medication, Moreover, dexamethasone was added for these patients.

The rest of the patients developed only mild to moderate COVID‐19 symptoms, andwas treated with antibiotic therapy (Augmentin), supportive therapy of vitamins C, D, zinc, and antipyretic medication like paracetamol (Table 1).

**Table 1:** Detailed information of the treated cases.

Case No.	Age	Gender	Medical Status regarding diabetes millets	Site	Presenting symptoms	Radiographic findings	Time from beginning of symptoms until surgery (days)
1	60	Male	Diabetic type 2	Left maxilla	Recurrent episodes of buccal space swelling, mobility of associated teeth	CBCT shows obliteration of the maxillary sinus on the affected site with necrotic bone islands	60
2	55	Male	Diabetic type 2	Right maxilla	Dental infection, teeth mobility	CBCT shows obliteration of both maxillary sinuses with necrotic bone islands and severe bone resorption involving a buccal bone plate of related teeth	60
3	39	Female	Diabetes‐like syndrome during COVID‐19 infection	Right maxilla	Severe headache with teeth mobility	CBCT shows obliteration of the maxillary sinus, thickening of ethmoidal sinus membrane with vertical bone loss around affected teeth with loss of buccal bone plate	60
4	57	Female	Diabetes‐like syndrome during COVID‐19 infection	Right and left maxilla	Headache and check swelling with teeth mobility	CBCT shows obliteration of both maxillary sinuses with necrotic bone islands and bone resorption of the buccal bone plate of the related teeth	120
5	65	Male	Diabetic type 2	Right maxilla	Teeth mobility with multiple sinuses over affected teeth	CBCT shows obliteration of both maxillary sinuses with bone destruction on the palatal and buccal side with necrotic bone islands	60
6	59	Male	Diabetes‐like syndrome during the COVID‐19 infection	Right maxilla	Severe pain with multiple sinuses with exposed yellowish bone with gum recession	CBCT shows obliteration of maxillary sinuses with necrotic bone and bone resorption of the buccal bone plate of the related teeth	50
7	50	Male	Nondiabetic	Left maxilla	Teeth mobility	CBCT shows obliteration of the maxillary sinus with vertical bone loss around affected teeth with loss of buccal bone plate	30
8	78	Female	Nondiabetic	Right maxilla	Teeth mobility	CBCT shows obliteration of the maxillary sinus with vertical bone loss around affected teeth with loss of buccal bone plate	120
9	35	Male	Nondiabetic	Right maxilla	Mobility of teeth with a history of losing upper first molar due to severe mobility, pus discharge from the palatal side	CBCT shows obliteration of the maxillary sinus, thickening of ethmoidal sinus membrane with vertical bone loss around affected teeth with loss of buccal bone plate	60
10	45	Female	Diabetic type 2	Right maxilla	Teeth mobility	CBCT shows obliteration of the maxillary sinus with vertical bone loss around affected teeth with loss of buccal bone plate	55
11	55	Male	Diabetic type 2	Right maxilla	Severe headache and mobility of teeth	CBCT shows obliteration of the maxillary sinus, thickening of ethmoidal sinus membrane with vertical bone loss around affected teeth with loss of buccal bone plate	50
12	38	Female	Diabetes‐like syndrome during COVID‐19 infection	Left maxilla	Dental infection that did not respond to any treatment, teeth with pus discharge from the left maxillary area close to the 1st molar	CBCT shows obliteration of the maxillary sinus, thickening of ethmoidal sinus membrane with vertical bone loss around affected teeth with loss of buccal bone plate	60
13	72	Male	Diabetic type 2	Right maxilla	Mobility of teeth	CBCT shows obliteration of the maxillary sinus with vertical bone loss around affected teeth with loss of buccal bone plate	70
14	62	Male	Diabetes‐like syndrome during COVID‐19 infection	Left maxilla	Multiple episodes of buccal and canine space infection resolved with antibiotic therapy and recur after cessation of treatment followed by the mobility of the related teeth	CBCT shows obliteration of the maxillary sinus, thickening of ethmoidal sinus membrane with vertical bone loss around affected teeth with loss of buccal bone plate	75
15	61	Female	Diabetic type 2	Right maxilla	Mobility of teeth	CBCT shows obliteration of the maxillary sinus, thickening of ethmoidal sinus membrane with vertical bone loss around affected teeth with loss of buccal bone plate	60

*Note*: No intracranial involvement was seen in all of these patients, but bilateral sinus involvement was seen in three patients.

Histopathological examination shows extensive necrosis with heavy mixed inflammatory cells infiltrating the surrounding focal aggregates of large and broad nonseptate fungal hyphae and spores, which are compatible with mucormycosis.

Two patients died after sudden cardiac arrest 2 weeks following surgical curettage; both had no history of diabetes, and one of them had 120 days delay from the symptoms onset of mucormycosis until he sought consult for treatment; the other one had a severe COVID‐19 infection.

## 4 DISCUSSION

COVID‐19 and its variants have caused a wide range of respiratory symptoms besides various other manifestations. COVID‐19 has been documented to have new associated symptoms and behaviors each day (Mañón et al., 2022; Mehta & Pandey, 2020; Song et al., 2020; Werthman‐Ehrenreich, 2021; Yadav et al., 2020).

In the last COVID‐19 surge in Iraq last July–September 2021, several of the affected unvaccinated patients had a severe type of respiratory infections that led to hypoxia and decreased oxygen saturation levels, hence necessitating admission to the intensive care unit.

In September 2020, Werthman‐Ehrenreich reported a case of 33‐year‐old female who had diabetic ketoacidosis and mucormycosis involving maxillary and ethmoidal air sinuses, and was associated with left eye proptosis and COVID‐19 (Werthman‐Ehrenreich, 2021). This was the first documented case in the literature showing the association between mucormycosis, COVID‐19, and diabetes (Werthman‐Ehrenreich, 2021).

In this research, most patients have diabetes or diabetic‐like syndrome. About half of them complained of a severe type of COVID‐19 infection and were given dexamethasone to relieve the cytokine storm. The combination of dexamethasone, which may lower a patient's immunity, and diabetes leads to the development of mucormycosis.

However, seven patients had mild COVID‐19 symptoms while three had no history of diabetes mellitus, but still developed mucormycosis. This finding showed that even mild COVID‐19 infection and absence of debilitating diseases could lead to post‐Covid 19 mucormycosis.

Mucormycosis is an aggressive, lethal fungal infection with rhinocerebral extension that may affect immunocompromised patients, (Mehta & Pandey, 2020) and the standard treatment protocol involves aggressive surgical debridement with antifungal therapy (Bakathir, 2006).

Even though it has a low incidence rate, ranging from 0.005 to 1.7 per million population, a significant increase in the number of diagnosed cases was seen a few months after the outbreak of the coronavirus pandemic (Deeplata et al., 2021). If the diagnosis is delayed, the mortality rate associated with mucormycosis can be doubled from 35% to 66%, so early diagnosis and treatment (surgical and medical) are essential (Mehta & Pandey, 2020). Despite all these facts, post‐COVID 19 mucormycosis manifests with slow progression and low mortality rate. The bony tissue showed a yellowish appearance with intact overlying mucosa.

Two patients were completely healed with no recurrence of the disease in a 4‐month follow‐up despite no antifungal liposomal amphotericin being used. This may indicate that surgical curettage, which removes all necrotic tissues and affected teeth, is the gold standard in managing post COVID‐19 mucormycosis. Accidental results with two patients were no liposomal amphotericin B was used, which influenced us to reduce the dose of liposomal amphotericin B from 5 to 1.5 mg/kg for the following five patients with the same successful results. Besides that, the average 2 months delay in referring these patients to the maxillofacial consultation clinics did not influence the survival rate; these findings may disagree with previous studies regarding the classical mucormycosis that spreads within a few days to end with a severe condition (Tran & Schmit, 2020). These findings may draw attention to the uniqueness of the post COVID‐19 mucormycosis characteristics, particularly its slow spread and low pathogenicity, which might be due to the immune system response being lowered by the medications used in treating COVID‐9. The opportunistic fungal infection has caused damage and necrosis, which is then slowed down due to the return of the patient's immunity to normal after controlling COVID‐19 and cessation of its medical treatment. Hence, it is only a transient episode unlike in classical mucormycosis, this is not the situation as the underlying cause is still ongoing (Tang et al., 2021).

COVID‐19 is considered a potential risk for developing sudden cardiac death. Several factors have been proposed to cause sudden cardiac death in post COVID‐19 patients, (Yadav et al., 2020) but the exact mechanism remains uncertain.

Anticoagulant drugs were used for COVID‐19 patients; however, they may develop a hypercoagulable state, (Mañón et al., 2022) which may contribute to the development of thromboembolic events. This may eventually make patients susceptible to sudden cardiac death and developing mucormycosis. Close monitoring of cardiac functions for these patients is crucial. However, further studies need to draw the relationship between post COVID‐19 mucormycosis and sudden cardiac death.

In conclusion, being highly suspicious of all patients affected with COVID‐19 is highly recommended to avoid the complications of diagnosing mucormycosis late. In addition, our knowledge and methods in diagnosing and treating classical mucormycosis should be modified regarding post COVID‐19 Mucormycosis.

## AUTHOR CONTRIBUTIONS

**Hassanien A. AL‐jumaily**: Conceptualization; methodology; intervention; and supervision. **Auday M. AL‐Anee**: Patients' follow‐up; validation; reviewing; and editing. **Ahmed F. Al‐Quisi**: writing original draft; data curation; formal analysis; and editing. All authors participated in discussing outcomes and the final revision of the manuscript, and all of them read and approved the final manuscript.

## CONFLICT OF INTEREST STATEMENT

The authors declare no conflict of interest.

## ETHICS STATEMENT

The protocol of this study was ethically approved by The Research Ethics Committee of the College of Dentistry, University of Baghdad (Project No.633122). Informed consent obtained from all patients.

## Data Availability

All the data of the research are included in this manuscript.
